# (*Z*)-2-Acetamido-3-(4-chloro­phen­yl)acrylic acid

**DOI:** 10.1107/S1600536809048041

**Published:** 2009-11-18

**Authors:** Qi-Jian Tian, Hui Ouyang, Chun-Lian Tian, Yong-Dong Jiang

**Affiliations:** a Key Laboratory of Hunan Forest Products and, Chemical Industry Engineering, Jishou University, Jishou 416000, People’s Republic of China

## Abstract

In the title compound, C_11_H_10_ClNO_3_, the mol­ecule consists of a benzene ring and an acetamido­acrylic acid unit on opposite sides of the C=C double bond. In the crystal, inter­molecular O—H⋯O and N—H⋯O hydrogen bonds assemble the mol­ecules into infinite two-dimensional ribbons. These ribbons are linked into a network by inter­molecular C—H⋯π contacts.

## Related literature

Derivatives of 2-acetamido-3-phenyl­acrylic acid are key inter­mates in the preparations of tanshinol (Wong *et al.* 1992 ▶; Xiao, *et al.* 2008*a* ▶), diaryl-3-hydr­oxy-2(5*H*)-furan­ones (Weber *et al.* 2002 ▶; Xiao *et al.* 2008*b* ▶) and benzyl­azauracil (Chen *et al.* 1993 ▶; Xiao, *et al.* 2008*c* ▶), which show anti-platelet aggregation, anti­fungal and anti­viral activities, respectively.
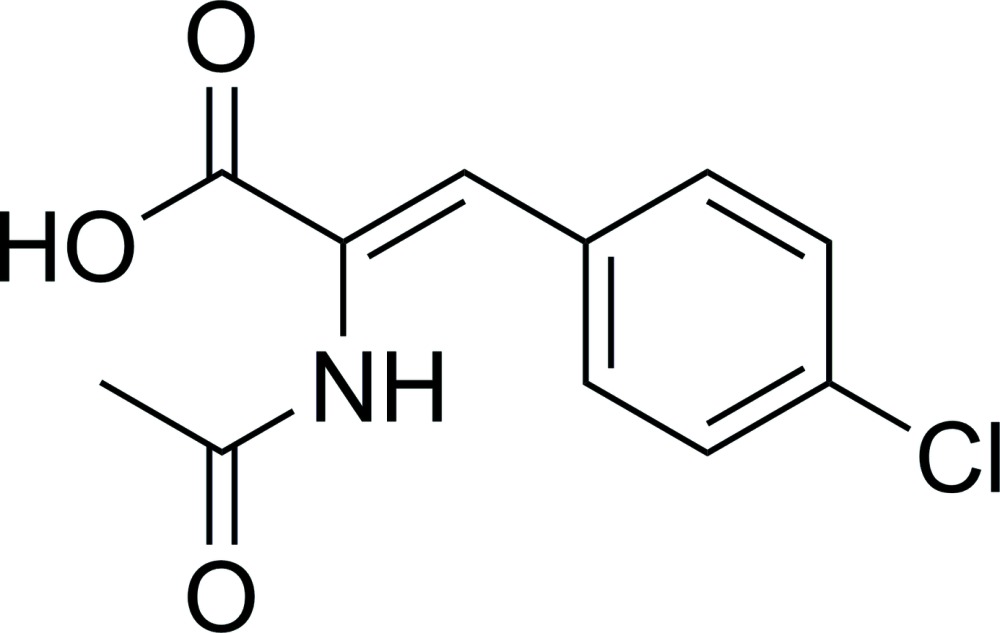


## Experimental

### 

#### Crystal data

C_11_H_10_ClNO_3_
*M* *_r_* = 239.65
Monoclinic, 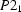

*a* = 6.2440 (12) Å
*b* = 7.5450 (15) Å
*c* = 11.813 (2) Å
β = 100.47 (3)°
*V* = 547.26 (19) Å^3^
*Z* = 2
Mo *K*α radiation
μ = 0.34 mm^−1^
*T* = 298 K
0.20 × 0.10 × 0.10 mm

#### Data collection

Bruker SMART APEX area-detector diffractometer
Absorption correction: multi-scan (*SADABS*; Sheldrick, 1996 ▶) *T*_min_ = 0.935, *T*_max_ = 0.967
1160 measured reflections
1060 independent reflections
895 reflections with *I* > 2σ(*I*)
*R*_int_ = 0.036

#### Refinement

*R*[*F* ^2^ > 2σ(*F* ^2^)] = 0.052
*wR*(*F* ^2^) = 0.229
*S* = 1.01
1060 reflections
148 parameters
1 restraint
H-atom parameters constrained
Δρ_max_ = 0.32 e Å^−3^
Δρ_min_ = −0.35 e Å^−3^

### 

Data collection: *SMART* (Bruker, 2007 ▶); cell refinement: *SAINT* (Bruker, 2007 ▶); data reduction: *SAINT*; program(s) used to solve structure: *SHELXS97* (Sheldrick, 2008 ▶); program(s) used to refine structure: *SHELXL97* (Sheldrick, 2008 ▶); molecular graphics: *SHELXTL* (Sheldrick, 2008 ▶); software used to prepare material for publication: *SHELXL97*.

## Supplementary Material

Crystal structure: contains datablocks global, I. DOI: 10.1107/S1600536809048041/bq2173sup1.cif

Structure factors: contains datablocks I. DOI: 10.1107/S1600536809048041/bq2173Isup2.hkl

Additional supplementary materials:  crystallographic information; 3D view; checkCIF report

## Figures and Tables

**Table 1:** Hydrogen-bond geometry (Å, °)

*D*—H⋯*A*	*D*—H	H⋯*A*	*D*⋯*A*	*D*—H⋯*A*
N1—H1⋯O1^i^	0.86	2.09	2.933 (7)	165
O2—H2*A*⋯O3^ii^	0.82	1.86	2.606 (7)	152
C3—H3⋯*Cg*1^iii^	0.93	2.85	3.523 (8)	130

Symmetry codes: (i) 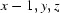; (ii) 
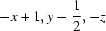
; (iii) 
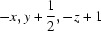
. *Cg*1 is the centroid of the C1–C6 ring.

**Fig. 1.**
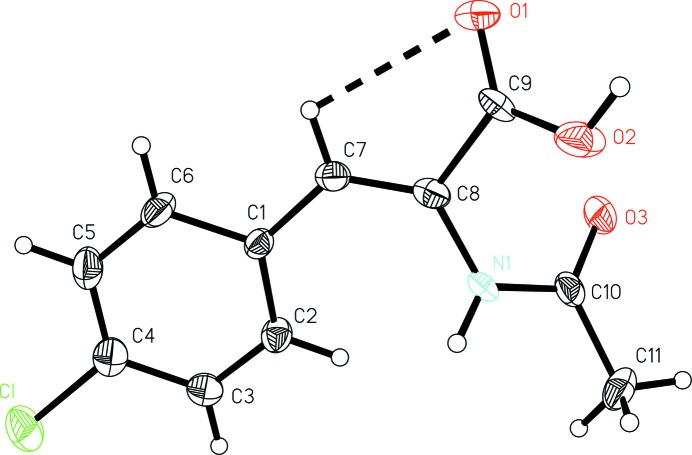
A view of (I), with the atom-numbering scheme and 30% probability displacement ellipsoids.

**Fig. 2.**
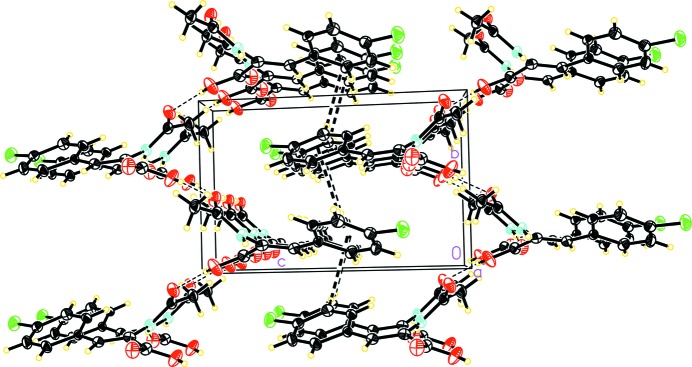
An infinite two-dimensional ribbon is formed through intermolecular O—H···O hydrogen bonds. Dashed lines indicate hydrogen bonds and solid dashed lines indicate C—H···π contacts.

## supplementary crystallographic information

### Comment

Derivatives of 2-acetamido-3-phenylacrylic acid are key intermates for tanshinol (Wong *et al.* 1992; Xiao, *et al.* 2008*a*), diaryl-3-hydroxy-2(5*H*)-furanones (Weber *et al.* 2002; Xiao *et al.* 2008*b*) and benzylazauracil (Chen *et al.* 1993; Xiao, *et al.* 2008*c*), which show anti-platelet aggregation, antifungal and antiviral activities, respectively. In the course of our work on screening for anticancers, we synthesized the title compound and herein reported its crystal structure.

In the title compound (I), the plane of benzene ring (with mean dieviation deviation of 0.0053 Å) and the plane of hydroxy acrylic moiety (with mean deviation of 0.0049 Å) make a dihedral angle of 18.001 (97) Å. The benzene ring and the carboxy group occur on opposite side of the C8═C9 double bond with torsion angle of 179.8 (4) ° (Fig. 1). Intermolecular O—H···O and N—H···O hydrogen bonds (Table 1) assemble the molecules into an infinite two-dimensional ribbon. This ribbons further form a network *via* C—H···pi contact (Fig. 2).

### Experimental

The mixture of alpha-acetoaminocinnamic acid (2.35 g, 10 mmol) in 0.5*M* HCl (60 mL) was refluxed for 3 h. The resulting mixture was allowed to cool to room temperature and the resulting precipitate was collected by filtration. The crude product was dissolved in EtOAc and twofold volume of petroleum was added carefully. Colorless blocks of (I) suitable for single-crystal structure determination was furnished after 2 d.

### Refinement

All H atoms were placed in geometrically idealized positions and constrained to ride on their parent atoms with C—H of 0.93 Å for the aromatic atoms and =CH groups, 0.96 Å for the CH_3_ groups, 0.82 Å for the OH groups and 0.86 Å for the NH groups. *U*_iso_(H) values were set at 1.2 times *U*_eq_(C) for aromatic C double bond C groups, 1.5 times *U*_eq_(C) for CH_3_ and 1.5 times *U*_eq_(O) for O—H groups. Because the absolute structure parameter is meaningless with a rather poor accuracy, the chemical absolute configuration could not be determined unambiguously

### Crystal data

**Table:**

C_11_H_10_ClNO_3_	*F*(000) = 248
*M**_r_* = 239.65	*D*_x_ = 1.454 Mg m^−^^3^
Monoclinic, *P*2_1_	Mo *K*α radiation, λ = 0.71073 Å
Hall symbol: P 2yb	Cell parameters from 775 reflections
*a* = 6.2440 (12) Å	θ = 1.9–24.7°
*b* = 7.5450 (15) Å	µ = 0.34 mm^−^^1^
*c* = 11.813 (2) Å	*T* = 298 K
β = 100.47 (3)°	Block, colorless
*V* = 547.26 (19) Å^3^	0.20 × 0.10 × 0.10 mm
*Z* = 2	

### Data collection

**Table:**

Bruker SMART APEX area-detector diffractometer	1060 independent reflections
Radiation source: fine-focus sealed tube	895 reflections with *I* > 2σ(*I*)
graphite	*R*_int_ = 0.036
φ and ω scans	θ_max_ = 25.2°, θ_min_ = 1.8°
Absorption correction: multi-scan (*SADABS*; Sheldrick, 1996)	*h* = 0→7
*T*_min_ = 0.935, *T*_max_ = 0.967	*k* = 0→9
1160 measured reflections	*l* = −14→13

### Refinement

**Table:**

Refinement on *F*^2^	Primary atom site location: structure-invariant direct methods
Least-squares matrix: full	Secondary atom site location: difference Fourier map
*R*[*F*^2^ > 2σ(*F*^2^)] = 0.052	Hydrogen site location: inferred from neighbouring sites
*wR*(*F*^2^) = 0.229	H-atom parameters constrained
*S* = 1.01	*w* = 1/[σ^2^(*F*_o_^2^) + (0.2*P*)^2^] where *P* = (*F*_o_^2^ + 2*F*_c_^2^)/3
1060 reflections	(Δ/σ)_max_ = 0.001
148 parameters	Δρ_max_ = 0.32 e Å^−^^3^
1 restraint	Δρ_min_ = −0.35 e Å^−^^3^

### Special details

**Table:**

Experimental. We have re-refined our data by using 'MERG 1' instruction to avoid Friedel opposites being merged. The absolute structure parameter is still meaningless, though the data/parameter (985/148) is higher than the former (895/148).
Geometry. All e.s.d.'s (except the e.s.d. in the dihedral angle between two l.s. planes) are estimated using the full covariance matrix. The cell e.s.d.'s are taken into account individually in the estimation of e.s.d.'s in distances, angles and torsion angles; correlations between e.s.d.'s in cell parameters are only used when they are defined by crystal symmetry. An approximate (isotropic) treatment of cell e.s.d.'s is used for estimating e.s.d.'s involving l.s. planes.
Refinement. Refinement of *F*^2^ against ALL reflections. The weighted *R*-factor *wR* and goodness of fit *S* are based on *F*^2^, conventional *R*-factors *R* are based on *F*, with *F* set to zero for negative *F*^2^. The threshold expression of *F*^2^ > σ(*F*^2^) is used only for calculating *R*-factors(gt) *etc*. and is not relevant to the choice of reflections for refinement. *R*-factors based on *F*^2^ are statistically about twice as large as those based on *F*, and *R*- factors based on ALL data will be even larger.

### Fractional atomic coordinates and isotropic or equivalent isotropic displacement parameters (Å^2^)

**Table:**

	*x*	*y*	*z*	*U*_iso_*/*U*_eq_	
Cl	−0.0425 (4)	0.7267 (4)	0.74675 (17)	0.0628 (8)	
C1	0.3337 (10)	0.6719 (8)	0.4546 (5)	0.0297 (14)	
N1	0.2032 (8)	0.7163 (9)	0.1907 (4)	0.0333 (13)	
H1	0.0860	0.6715	0.2071	0.06 (3)*	
O1	0.7621 (7)	0.6067 (9)	0.2131 (5)	0.0529 (16)	
C2	0.1323 (11)	0.7587 (9)	0.4407 (6)	0.0360 (16)	
H2	0.0726	0.8045	0.3688	0.043*	
O2	0.4735 (8)	0.5437 (10)	0.0782 (5)	0.0579 (17)	
H2A	0.5657	0.5193	0.0393	0.087*	
C3	0.0201 (13)	0.7786 (10)	0.5294 (6)	0.0422 (18)	
H3	−0.1134	0.8368	0.5177	0.051*	
O3	0.3510 (8)	0.9179 (9)	0.0890 (4)	0.0496 (15)	
C4	0.1089 (13)	0.7102 (11)	0.6378 (6)	0.0436 (18)	
C5	0.3095 (13)	0.6317 (12)	0.6558 (6)	0.0480 (19)	
H5	0.3701	0.5915	0.7291	0.058*	
C6	0.4220 (11)	0.6116 (11)	0.5683 (6)	0.0424 (18)	
H6	0.5582	0.5577	0.5825	0.051*	
C7	0.4613 (10)	0.6378 (10)	0.3662 (6)	0.0337 (14)	
H7	0.6012	0.5950	0.3924	0.040*	
C8	0.4067 (10)	0.6591 (10)	0.2525 (6)	0.0352 (15)	
C9	0.5701 (10)	0.6022 (10)	0.1796 (6)	0.0372 (16)	
C10	0.1874 (11)	0.8392 (10)	0.1065 (5)	0.0363 (15)	
C11	−0.0380 (12)	0.8731 (13)	0.0403 (6)	0.050 (2)	
H11A	−0.0295	0.9462	−0.0252	0.075*	
H11B	−0.1227	0.9323	0.0891	0.075*	
H11C	−0.1059	0.7624	0.0149	0.075*	

### Atomic displacement parameters (Å^2^)

**Table:**

	*U*^11^	*U*^22^	*U*^33^	*U*^12^	*U*^13^	*U*^23^
Cl	0.0756 (15)	0.0751 (15)	0.0438 (10)	−0.0037 (13)	0.0266 (10)	−0.0043 (11)
C1	0.028 (3)	0.032 (3)	0.026 (3)	−0.003 (3)	−0.001 (2)	0.002 (2)
N1	0.027 (3)	0.045 (3)	0.032 (2)	0.000 (3)	0.016 (2)	0.004 (3)
O1	0.025 (2)	0.077 (4)	0.057 (3)	0.003 (3)	0.005 (2)	0.000 (3)
C2	0.041 (4)	0.033 (4)	0.034 (3)	0.008 (3)	0.006 (3)	0.004 (3)
O2	0.033 (2)	0.084 (4)	0.058 (3)	−0.001 (3)	0.012 (2)	−0.034 (4)
C3	0.037 (4)	0.046 (4)	0.045 (4)	0.004 (3)	0.011 (3)	0.000 (3)
O3	0.047 (3)	0.063 (4)	0.041 (3)	−0.013 (3)	0.015 (2)	0.014 (3)
C4	0.051 (4)	0.038 (4)	0.040 (4)	−0.008 (4)	0.003 (3)	−0.002 (3)
C5	0.059 (5)	0.054 (5)	0.030 (3)	−0.005 (4)	0.005 (3)	0.006 (3)
C6	0.038 (4)	0.046 (4)	0.039 (4)	0.012 (3)	−0.004 (3)	0.008 (3)
C7	0.027 (3)	0.035 (3)	0.040 (3)	0.002 (3)	0.006 (2)	0.002 (3)
C8	0.029 (3)	0.034 (3)	0.045 (4)	0.000 (3)	0.014 (3)	0.003 (3)
C9	0.033 (3)	0.044 (4)	0.041 (4)	−0.004 (3)	0.022 (3)	−0.002 (3)
C10	0.040 (3)	0.044 (4)	0.029 (3)	0.005 (3)	0.016 (3)	−0.003 (3)
C11	0.054 (4)	0.057 (5)	0.034 (3)	0.011 (4)	−0.006 (3)	0.006 (4)

### Geometric parameters (Å, °)

**Table:**

Cl—C4	1.734 (8)	C3—H3	0.9300
C1—C2	1.401 (9)	O3—C10	1.231 (9)
C1—C6	1.430 (9)	C4—C5	1.367 (12)
C1—C7	1.446 (9)	C5—C6	1.360 (11)
N1—C10	1.350 (9)	C5—H5	0.9300
N1—C8	1.413 (9)	C6—H6	0.9300
N1—H1	0.8600	C7—C8	1.334 (11)
O1—C9	1.193 (8)	C7—H7	0.9300
C2—C3	1.370 (11)	C8—C9	1.512 (8)
C2—H2	0.9300	C10—C11	1.502 (10)
O2—C9	1.316 (9)	C11—H11A	0.9600
O2—H2A	0.8200	C11—H11B	0.9600
C3—C4	1.398 (11)	C11—H11C	0.9600
			
C2—C1—C6	116.3 (6)	C5—C6—H6	119.5
C2—C1—C7	126.8 (6)	C1—C6—H6	119.5
C6—C1—C7	116.9 (6)	C8—C7—C1	129.2 (6)
C10—N1—C8	121.9 (6)	C8—C7—H7	115.4
C10—N1—H1	119.0	C1—C7—H7	115.4
C8—N1—H1	119.0	C7—C8—N1	126.8 (6)
C3—C2—C1	122.3 (6)	C7—C8—C9	117.6 (6)
C3—C2—H2	118.8	N1—C8—C9	115.4 (6)
C1—C2—H2	118.8	O1—C9—O2	125.3 (6)
C9—O2—H2A	109.5	O1—C9—C8	123.0 (6)
C4—C3—C2	119.2 (7)	O2—C9—C8	111.7 (5)
C4—C3—H3	120.4	O3—C10—N1	120.2 (6)
C2—C3—H3	120.4	O3—C10—C11	123.9 (7)
C3—C4—C5	120.1 (7)	N1—C10—C11	115.9 (6)
C3—C4—Cl	118.3 (6)	C10—C11—H11A	109.5
C5—C4—Cl	121.6 (6)	C10—C11—H11B	109.5
C4—C5—C6	121.0 (7)	H11A—C11—H11B	109.5
C4—C5—H5	119.5	C10—C11—H11C	109.5
C6—C5—H5	119.5	H11A—C11—H11C	109.5
C5—C6—C1	121.0 (6)	H11B—C11—H11C	109.5
			
C6—C1—C2—C3	−2.8 (10)	C6—C1—C7—C8	170.8 (8)
C7—C1—C2—C3	177.5 (7)	C1—C7—C8—N1	−2.2 (13)
C1—C2—C3—C4	0.0 (11)	C1—C7—C8—C9	−176.5 (7)
C2—C3—C4—C5	2.9 (12)	C10—N1—C8—C7	134.8 (8)
C2—C3—C4—Cl	−176.7 (6)	C10—N1—C8—C9	−50.8 (9)
C3—C4—C5—C6	−2.9 (13)	C7—C8—C9—O1	−30.5 (11)
Cl—C4—C5—C6	176.6 (6)	N1—C8—C9—O1	154.6 (8)
C4—C5—C6—C1	0.0 (12)	C7—C8—C9—O2	147.7 (8)
C2—C1—C6—C5	2.8 (11)	N1—C8—C9—O2	−27.2 (10)
C7—C1—C6—C5	−177.5 (7)	C8—N1—C10—O3	−7.2 (11)
C2—C1—C7—C8	−9.5 (12)	C8—N1—C10—C11	174.0 (6)

### Hydrogen-bond geometry (Å, °)

**Table:**

*D*—H···*A*	*D*—H	H···*A*	*D*···*A*	*D*—H···*A*
N1—H1···O1^i^	0.86	2.09	2.933 (7)	165
O2—H2A···O3^ii^	0.82	1.86	2.606 (7)	152
C3—H3···Cg1^iii^	0.93	2.85	3.523 (8)	130

Symmetry codes: (i) *x*−1, *y*, *z*; (ii) −*x*+1, *y*−1/2, −*z*; (iii) −*x*, *y*+1/2, −*z*+1.
